# Exploring research capacity and culture of allied health professionals: a mixed methods evaluation

**DOI:** 10.1186/s12913-022-07480-x

**Published:** 2022-01-17

**Authors:** Terry Cordrey, Elizabeth King, Emma Pilkington, Katie Gore, Owen Gustafson

**Affiliations:** 1 Oxford Allied Health Professions Research & Innovation Unit, Oxford University Hospitals NHS Foundation Trust, OX3 9DU Oxford, UK; 2 Centre for Movement, Occupational and Rehabilitation Sciences, Faculty of Health and Life Sciences, Oxford Brookes University, OX3 0BP Oxford, UK

**Keywords:** Mixed methods, Research capacity, Allied health professional, Research culture, Research activity

## Abstract

**Background:**

Despite the myriad benefits of research to patients, professionals, and organisations, fewer than 0.1% of the Allied Health Professions workforce are employed in clinical academic roles. Identified barriers include a lack of role modelling, management support, funding, and availability of clinical academic roles. Research capacity building is critical to improving Allied Health Professional research capability. The aim of this evaluation was to explore the current research capacity and culture of Allied Health Professionals to inform future tailored research capacity building strategies at a local level.

**Methods:**

A mixed methods evaluation of research capacity and culture was conducted within the Allied Health Professions department of a large National Health Service Foundation Trust using an online research capacity and culture questionnaire, followed by focus groups. Staff were recruited using a purposive method with the questionnaire and subsequent focus groups completed between July and September 2020. Data from the questionnaire was analysed using simple descriptive statistics and after inductive coding, focus group data was analysed thematically.

**Results:**

93 out of 278 staff completed the questionnaire and 60 staff members attended seven focus groups. The research capacity and culture survey reported the department’s key strength as promoting clinical practice based on evidence (median=8, range=6-9). A key reported weakness of the department was insufficient resources to support staff research training (med=4, 3-6). Respondents considered themselves most skilled in finding relevant literature (med=6, 5-8) and least skilled at securing research funding (med=1, 1-2). Greater than half of the respondents (n=50) reported not currently being involved with research. Five themes were identified from the focus groups: empowerment; building research infrastructure; fostering research skills; access for all; and positive research culture.

**Conclusions:**

Allied Health Professionals recognise the benefits of research at teams and departmental level, but marginally at an individual level. Local research capacity building strategies should aim to address the role, responsibilities and barriers to Allied Health Profession research development at an individual level. To ensure all staff can engage, research infrastructure and empowerment are essential.

**Supplementary Information:**

The online version contains supplementary material available at 10.1186/s12913-022-07480-x.

## Background

Research active healthcare organisations have a lower risk-adjusted mortality rate, higher levels of patient experience, and better staff recruitment and retention compared to those with a lower research profile [1–4]. The benefits of research extend beyond healthcare; between 2016 and 2019, the National Institute of Health Research (NIHR) contributed an estimated £8bn to the UK economy and generated over 47,000 jobs through its clinical research network activity [5]. Despite the myriad benefits, research is still viewed as a luxurious pursuit reserved for those who work outside of busy clinical roles [6]. This view is potentially re-enforced by a sustained reduction in the capacity of NHS staff to undertake research. Between 2004 and 2017, the proportion of consultant physicians working in clinical academic roles in the NHS has reduced from 7.5 to 4.2% [7]. For Allied Health Professionals (AHP), the historical position is not known, but the proportion of AHPs currently working in clinical academic roles is approximately 0.1% [8]. There is no shortage of desire amongst AHPs to engage in research activity, but numerous barriers are cited preventing the realisation of these ambitions [9]. The lack of structure, funding, and access to clinical academic career pathways are the most commonly cited barriers to the growth of AHP research capacity [10]. Contributing to this issue is an absence of strong role models, a lack of expectation of academic achievement, and challenges in ‘being released’ from a clinical role [11]. In England, the consequences of these barriers are well illustrated through the findings of a 10-year review from a the NIHR national programme of integrated clinical academic training. Despite relatively good AHP uptake into early research career awards, progression into doctoral-level study and attaining post-doctoral fellowships was poor [12]. The latter has been identified as a key enabling mechanism to achieve a successful clinical academic career [13]. Insufficient supply of AHPs into post-doctoral roles perpetuates the current position of a lack of senior clinical academic AHP leaders and decision-makers, which potentially serves to inhibit future AHP research capacity building strategies [14].

Research capacity building (RCB) is a key determinant in improving research quality and its translation to clinical practice [15]. Defined as, “*a process of individual and institutional development which leads to higher levels of skills and greater ability to perform useful research”*, effective RCB requires an integrated strategic and policy-informed approach that targets individual, organisational and system levels [16]. Several key elements are thought to underpin successful AHP RCB efforts. These include strong strategic leadership, effective partnerships between health and academic institutions, funded research career pathways, placing value on research and ensuring good support mechanisms for individual researchers [15, 17].

The only notable large scale RCB effort including AHPs has been delivered through the NIHR integrated clinical academic (ICA) training programme. This is a national programme focused on building research capacity and capability through individual training awards [18]. Professions eligible for these awards, including AHPs, form a population of over a half a million staff accounting for over 80% of all qualified health care professionals (HCP) in England [19] The volume of eligible professionals for this pathway breeds a high level of competition for relatively low amounts of funding proportionate to the size of the qualified non-medical workforce [20]. An absence of detailed evaluation specifically focused on AHPs means it is difficult to determine the extent to which AHP research capacity has benefited from this national programme. When smaller-scale RCB efforts have focused discretely on AHPs, outcomes have been favourable in terms acquisition of research skills, experience and outputs [21, 22]. Local RCB programmes are context-specific in their approach and perhaps can achieve (1) greater access to research development funds, and (2) greater congruence between local health and care system research priorities and RCB strategies to address them [12]. The aim of the project was to evaluate the current research capacity and culture (RCC) among AHPs working at a large university teaching NHS Foundation Trust, to inform the future development of tailored research capacity building strategies.

## Methods

This mixed methods evaluation of RCC was undertaken within an AHP department at a large university teaching NHS Foundation Trust in the UK. The department comprises 278 staff employed as dietitians, occupational therapists, physiotherapists, speech and language therapists and support staff. Services are provided in acute and sub-acute adult clinical specialties over four hospital sites. All staff within the AHP department were invited to participate in an online RCC questionnaire followed by structured focus groups to enable in-depth exploration of the themes that emerged from the RCC questionnaire results. This manuscript has been prepared according to the COREQ reporting guidelines [23], with the checklist available in Supplementary Material 1. The project proposal was submitted to the NHS Trust’s research and development office and was classified as a service evaluation not requiring ethical approval (Ulysses ID: 6309). Good research governance was observed throughout with participant information provided and informed consent gained for both data collection stages. Participants were made aware of their right to withdraw from the study at any time and were assured that any data published would be anonymised.

### Research capacity and culture tool

The RCC tool is a valid and reliable questionnaire that measures indicators of research capacity and culture at individual, team and organisation domains [24]. It can be used for undertaking research training needs analysis and to plan and evaluate research capacity building [25]. For the purposes of our evaluation, the “organisation” domain within the tool was defined as the AHP department and the “team” domain as the clinical specialty team that the participants worked within. To avoid participant identification during data analysis, further information about job role, grade and specific work location was not sought.

The survey was administered using a secure online platform (Microsoft Forms) and was made available between 8th July and 2nd August 2020. A multifaceted approach to participant recruitment was adopted; all staff within the department were invited by email to complete the survey and were sent reminder emails periodically. The survey was widely promoted using posters in staff rest areas, in departmental meetings, and notifications in the departmental staff bulletin.

### Focus group

The results of the RCC, in particular the motivators and barriers to research engagement, were used to construct a topic guide for the focus group. This was developed using a five-step framework to ensure methodological robustness [26]. The topic guide was piloted with two members (EP and KG) of the research team and subsequently revised allowing for the exploration of anticipated themes but also for the development of new themes (Supplementary Material 2).

 Focus group participants were recruited via email communication and a snowballing technique across the department. Focus groups were held with a minimum of four and a maximum of 12 participants per group in the department. Data saturation is not purely aligned with thematic analysis but was considered in the planning of the groups to allow for a sufficient sample [27]. The focus groups were led by members of the research team (OG and EK) with experience and training in qualitative research methods. Each focus group had a primary interviewer, and then one or two secondary interviewer/s who completed reflexive notes. Interviewees had varying degrees of working relationships with respondents. Audio recordings were transcribed verbatim and anonymised.

### Data analysis

The RCC questionnaire results were analysed using descriptive statistics. Likert-scale items within the RCC tool were summarised by the median and Inter Quartile Range (IQR), as a conventional method for ordinal data analysis, with all other items presented as numbers and percentages. Thematic analysis was chosen to describe both ‘implicit and explicit ideas’ from the focus group data [28]. Analysis of interview transcripts was supported by NVivo 12 (NVivo qualitative data analysis software; QSR International Pty Ltd. Version 12). EK examined the data and developed a coding tree of five parent and 30 child nodes. Through peer review with OG and TC, themes were developed and agreed upon.

## Results

A total of 93 staff completed the questionnaire, which represents a response rate of 33.4% of the 278 staff pool targeted. The largest group of respondents were physiotherapists (n= 44, 47.3%), with speech and language therapists (n=10, 20.8%) and support staff (n=6, 6.5%) recording the lowest number of responses, as outlined in Table 1. Occupational therapists (25.9%) and support staff (16.2%) were the lowest responders, as a proportion of their profession compared to speech and language therapists (50%), dietitians (42.9%) and physiotherapists (41.5%). Although 37.6% (n= 35) of respondents had undertaken a postgraduate degree, only three (3.2%) had completed or were currently enrolled on a formal research training degree programme.

**Table 1:** Participant characteristics

Profession	n (%)	Highest professional qualification	n (%)
Dietitian	18 (19.4)	No degree	0
Occupational Therapist	15 (16.1)	Certificate/diploma	4 (4.3)
Physiotherapist	44 (47.3)	Undergraduate	51 (54.8)
Speech & Language Therapist	10 (10.8)	Postgraduate	35 (37.6)
Support worker	6 (6.5)	Doctoral	3 (3.2)
**Clinical specialty of respondents**			**n (%)**
Acute, general and geriatric medicine			14 (15.05)
Cancer surgery and rehabilitation			12 (12.90)
Critical care			14 (15.05)
Diabetes/endocrinology			2 (2.15)
General and specialist surgery			7 (7.53)
Management			2 (2.15)
Neurology/Neurosurgery			4 (4.30)
Neurological and stroke rehabilitation			20 (21.51)
Clinical nutrition			4 (4.30)
Obstetrics & Gynaecology			5 (4.30)
Renal medicine			6 (4.30)
Respiratory medicine			6 (6.45)

Following the completion of the questionnaire, 60 staff attended seven focus groups across four hospital sites; consisting of 34 physiotherapists; 11 occupational therapists; one speech and language therapist; nine dieticians and five support staff. Focus group durations ranged from 47 to 61 minutes, with six to 12 staff attending each group.

### RCC

As illustrated in Table 2, participants reported the departments strengths to include promoting clinical practice based on evidence (med=8, 6-9), ensuring planning is guided by evidence (med=7, 4.25-8), encouraging research activities relevant to practice (med=7, 5-8), and having senior managers that support research (med=7, 5-8). In contrast, the reported weaknesses included having software programmes for analysing research data (med=2, 1-4.5), having funds, equipment or administrative support for research activities (med=3, 2-5), and accessing external funding for research (med=3, 1.75-5). Further reported weaknesses included ensuring staff career pathways are available in research (med=4, 2-5), having consumers involved in research (med=4, 2-7), having mechanisms to monitor research quality (med= 4, 1-6), and having adequate resources to support staff research training (med=4, 3-6). Similar areas of strengths and weaknesses were also reported in the team domain (Table 3).

**Table 2:** RCC results – Department

Item	Description The Therapy Department…	Median (IQR)	Unsure n (%)
i	…has adequate resources to support staff research training	4 (3-6)	17 (18)
ii	…has funds, equipment or admin to support research activities	3 (2-5)	23 (25)
iii	…has a plan or policy for research development	5 (2.5-7)	26 (28)
iv	…has senior managers that support research	7 (5-8)	5 (5)
v	…ensures staff career pathways are available in research	4 (2-5)	18 (19)
vi	…ensures organisation planning is guided by evidence	7 (4.25-8)	11 (12)
vii	…has consumers involved in research	4 (2-7)	46 (49)
viii	…accesses external funding for research	3 (1.75-5)	49 (53)
ix	…promotes clinical practice based on evidence	8 (6-9)	5 (5)
x	…encourages research activities relevant to practice	7 (5-8)	11 (12)
xi	…has software programs for analysing research data	2 (1-4.5)	62 (67)
xii	…has mechanisms to monitor research quality	4 (1-6)	48 (52)
xiii	…has identified experts accessible for research advice	5 (2-6.5)	38 (41)
xiv	…supports a multidisciplinary approach to research	5 (4-8)	24 (26)
xv	…has regular forums/bulletins to present research findings	5 (2-6)	12 (13)
xvi	…engages external parties (e.g. universities) in research	5 (3-7)	27 29)
xvii	…supports applications for research scholarships/degrees	5 (2-7)	30 (32)
xviii	…supports the peer-reviewed publication of research	6 (4-7)	37 (40)

**Table 3:** RCC results – Team

Item	Description My team…	Median (IQR)	Unsure n (%)
i	…has adequate resources to support staff research training	3 (1.5-6)	10 (11)
ii	…has funds, equipment or admin to support research activities	2 (1-5)	21 (23)
iii	…does team level planning for research development	3.5 (1-6)	11 (12)
iv	…ensures staff involvement in developing that plan	5 (1-7)	13 (14)
v	…has leaders that support research	7 (5-10)	5 (5)
vi	…provides opportunities to get involved in research	5 (2-7)	10 (11)
vii	…does planning that is guided by evidence	7 (5-8)	8 (9)
viii	…has consumer involvement in research activities/planning	2 (1-5)	35 (38)
ix	…has applied for external funding for research	2 (1-7)	33 (35)
x	…conducts research activities relevant to practice	5 (2-8)	8 (9)
xi	…supports applications for research scholarships/degrees	5 (1.75-8)	25 (27)
xii	…has mechanisms to monitor research quality	2 (1-6)	30 (32)
xiii	…has identified experts accessible for research advice	4 (1-7)	22 (24)
xiv	…disseminates research results at research forums/seminars	5 (3.7.5)	10 (11)
xv	…supports a multidisciplinary approach to research	6 (4-8)	16 (17)
xvi	…has incentives and support for mentoring activities	4 (1-6)	19 (20)
xvii	…has external partners (e.g. Universities) engaged in research	3 (1-7.25)	25 (27)
xviii	…supports the peer-reviewed publication of research	5 (1-8)	25 (27)
xix	…has software available to support research activities	1 (1-3)	43 (46)

As outlined in Table 4, participants considered themselves most skilled in finding relevant literature (med=6, 5-8) and critically reviewing the literature (med=6, 5-7.75). However, they considered themselves to be least skilled in securing research funding (med= 1, 1-2), submitting an ethics application (med=1, 1-3), writing a research protocol (med=2, 1-5), writing a publication for peer review (med=2, 1-5), and providing advice to less experienced researchers (med=2, 1-4). Greater than half of the respondents (n=50) were not currently undertaking any research activity and 58 (62%) had not completed any research outputs in the past 12 months (Supplementary Material 3, Table S1). Although 29 (31%) of respondents had research related activities as part of their role description, only six had dedicated time to do research and five reported access to research supervision (Supplementary Material 3, Table S1).

**Table 4:** RCC results – Individual

Item	Description I have skills in…	Median (IQR)	Unsure n (%)
i	…finding relevant literature	6 (5-8)	2 (2)
ii	…critically reviewing the literature	6 (5-7.75)	3 (3)
iii	…using a computer referencing system (e.g. Endnote)	4 (1-6)	9 (10)
iv	…writing a research protocol	2 (1-5)	6 (6)
v	…securing research funding	1 (1-2)	10 (11)
vi	…submitting an ethics application	1 (1-3)	9 (10)
vii	…designing questionnaires	5 (2.5-7)	7 (8)
viii	…collecting data e.g. surveys, interviews	5.5 (3-7)	5 (5)
ix	…using computer data management systems	3 (1-5.75)	7 (8)
x	…analysing qualitative research data	4 (1-5)	4 (4)
xi	…analysing quantitative research data	4 (1-5)	3 (3)
xii	…writing a research report	3 (1.5-6)	6 (6)
xiii	…writing for publication in peer reviewed journals	2 (1-5)	10 (11)
xiv	…providing advice to less experienced researchers	2 (1-4)	5 (5)

The most common barriers to participation in research were a lack time, funding, suitable role backfill, administrative support, other work taking priority, lack of individual skill, fear of getting it wrong and a desire for a work/life balance. In contrast, individual motivators to undertake research were to develop skills, advance career, increase job satisfaction, keep the brain stimulated and increase credibility. Further details are available in Table S2 and S3 (Supplementary Material 3).

### Focus Groups

Five grouped themes were identified from the focus groups: empowerment; building research infrastructure; fostering research skills; access for all; and positive research culture. Table 5 displays selected quotes to support the five themes.

**Table 5:** Grouped themes and selected quotes from the focus groups

Empowerment	
Endorsement to engage	‘…so actually having that full permission… will make a big difference in people actually participating in research.’
Overcoming competing factors	‘I know there will be that pressure the wards are full, and there is a big amount of caseload.’
Balancing workload priorities	‘It is difficult to step back and prioritise time for non-clinical things like research’
Dispelling personal and external guilt	‘…almost guilt from having not done the clinical or a guilt from how patients or families might feel, or how their colleagues might feel being left?’
**Building research infrastructure**	
Strong mentorship with accountability	‘Just having someone that can help you know walk you through that process…’
Breadth of communication channels	‘…someone having dedicated time to meet …whether it’s just emailing them or sitting down with them.’
Establishment of a resource repository	‘I think having a central place for research…. it would be nice to know where to go and how to access that.’
Nurturing collaborations internally and externally	‘So I think it is building those bridges between the disciplines together.’
**Fostering research skills**	
Acquiring research skills	‘…I think the opportunity to get involved with little bits, see if you do like it and learn how to do…’
Scale of skills across the research process	‘…writing a paper…’; ‘…ethics…’; ‘…presenting…creation of a paper…’
Formulating the right research questions	‘So right from setting your question which is complex enough sometimes, and having that so you don’t get it wrong at the beginning…’
Development of grassroot to advanced skills	‘The poster, the case study? It doesn’t necessarily have to be a big study does it?
Ownership of research skills and outputs	‘That’s the hardest thing… I went to a consultant with an idea and it got snatched, and I helped collect data and got funding, and got zero mention…’
**Access for all**	
Inclusivity for all staff	‘…make decisions what would help people from the time they start in the trust to the time where they become more senior researchers.’
Flexibility for staff with varying work patterns	‘And something less dependent on potentially rotating.’
Formal clinical academic pathways versus research engagement	‘So a clinical academic pathway as a post where you have protected time for research and training…’; ‘…having the exposure and opportunity to grow in particular areas of research’
**Positive research culture**	
Strengthening staff recruitment and retention	‘… it will only help with staff retention, for people to grow with that…’
Underpinning staff development and enriching staff experience	‘I think it helps develop you as a physio. If you don’t ever do research to change things you would still do things the way people did 50 years go.’
Showcasing local research profiles and priorities	‘…contributing to the physiotherapy department branding…. putting us out on the map….the centre of excellence for AHP research’
Ensuring evidence-based practice	‘Science is changing all the time so you are going to need an evidence based practice…’
Supporting commissioning and operational provision	‘I think there are huge benefits, that’s how you illicit change, isn’t it? It’s how you get to influence your service… to control future improvements in care essentially.’

Many staff identified the need to be granted permission to engage with research and to do this, it would require a cultural shift to dispel feelings of guilt and therefore prioritise research in conjunction with competing clinical workload factors. This suggests research is not seen as routine activity within the department thus requiring the development of a research infrastructure. Many staff described this as twofold: a pool of mentors with suitable experience to provide direction, accountability and have protected time in their role to do this; and secondly, a resource repository to support training and networking needs. Beyond the infrastructure, the development and application of research skills is required as it is suggested to date staff have not been exposed to many research skills. Similarly, it is suggested staff do not lead on the research process.

Many staff described the internal and external benefits of fostering a positive research culture. This includes staff recruitment and retention; strengthening commissioning; and profiling the department through dissemination of high-quality research. Internally, some staff described interest for dedicated clinical academic roles while others expressed interest in less formal research opportunities, such as time limited projects.

Table 6 displays the grouped themes from the focus groups aligned to the domains of the RCC. At the individual level only three themes were included, however all themes were recognised by the team and organisation domains.

**Table 6:** Themes aligned to RCC domains

	Individual	Team	Organisation
**Empowerment**	Guilt, prioritisation	Permission, prioritisation	No time – lack of job planning for research
**Building research infrastructure**		Training	Mentorship, role modelling, access to training, resources
**Fostering research skills**		Education	Exposure to skills/experience
**Allowing access for all**	Engagement	EBP within team	Formal CAC pathway Limitations in post design/structure - rotations/part-time
**Positive research culture**	Influences individual practice	Enhancing profile/recruitment	Enhancing profile/recruitment

## Discussion

The findings from this mixed methods evaluation of AHP RCC suggest that local RCB strategies need to address the challenges within all three domains and not exclusively the organisation domain as has been the focus of previous studies [29]. The resulting thematic model identified five key themes on which to develop the RCB strategy: empowerment; building research infrastructure; fostering research skills; access for all; and a positive research culture. These themes are consistent with those previously published [14], however despite all themes correlating with the three domains of the RCC tool, there are very few included within the individual domain. Several recently published studies identifying barriers to the development of AHP research capacity have almost exclusively focused on the team and organisation domains of the RCC tool [25, 30] with very little discussion to date of the role, responsibility and barriers to AHP research development at an individual level.

At an organisational and team level this study found that a lack of financial resources, dedicated career pathways and academic mentorship created the most significant barriers to RCB. Additionally, it suggests that future local RCB strategies should include a department wide education programme delivered centrally.

A lack of financial resources is consistent with the previously published literature [31], with healthcare organisations placing an increasing emphasis on efficiency of healthcare provision in the context of wider financial constraints and competing pressures [32]. To ensure resources are allocated to research training and support, AHP research needs to be embedded in the organisations wider vision and strategy [33, 34]. Local RCB building strategies need to be designed to maximise efficient use of resources, and clearly support the organisations overall objectives. The inclusion of AHP research within an organisation’s strategy will help to empower staff to prioritise research activity. This is important, as despite the RCC demonstrating that senior managers were supportive of research and encouraged clinical practice based on evidence, staff felt that they needed permission to prioritise research activity and manage those competing factors.

As with several previous studies, the lack of a clinical academic career pathway for AHPs was identified as a key barrier to RCB [35]. Despite national clinical academic training programmes for AHPs being established, this is not being widely adopted at a local level [36]. A key component of future local RCB strategies requires clear job planning, development and sustainability of a clinical academic career pathway for AHPs, to ensure that there are options beyond the highly competitive and limited number of opportunities at a national level [37, 38] In addition to formal clinical academic pathways, staff are interested in developing grassroots research skills and engaging with research without pursuing a formal clinical academic career. Irrespective of the individual career aims, research skills should be seen as complementary to clinical activity rather than being seen as strictly separate silos. Job planning and evaluation as part of local RCB strategies also needs to consider the challenges to participation and engagement faced by part time or rotational staff, to ensure they are supported in achieving their research goals. Accounting for individual career aims and current working arrangements in RCB strategies will create a positive research culture, and help to improve staff recruitment and retention [10].

It is unsurprising that a department without a clinical academic career pathway has a lack of research and academic mentorship for staff, which was subsequently perceived as a barrier to research participation. Development of mentorship opportunities for staff needs to be a key priority for RCB strategies as it has been recognised as a fundamental requirement of research infrastructure and early career researcher development [39]. Partnerships and collaborations with local higher education institutes can not only provide the opportunities for academic and research mentorship, but can also further progress research infrastructure and clinical academic career pathways through jointly funded positions [7]. As improved research capacity becomes established within the department, research ownership and leadership should be developed, raising the profile of the department’s research activity and opportunities for future collaborations.

Delivering research education to enable the application of evidence-based practice is a key thread across the team domain. As research should be seen as a core part of AHP job roles, the use of educational frameworks could support the combination of clinical and academic training[40]. The Council for Allied Health Professions Research has published a framework detailing levels of competencies from awareness to expert which would support the non-linear development of research skills and could be used to support education programme development [41]. The lack of established research infrastructure within the department means that research training would be better delivered centrally as opposed to within separate individual teams.

At an individual level, several staff highlighted feelings of guilt when prioritising research over their clinical work. This overwhelming focus on clinical work extends beyond a potential under appreciation of the benefits of research at an organisation and patient level, to a lack of understanding of the benefits to them as individuals. Although some staff identified having skills that enable them to undertake some individual research activities such as finding and critiquing literature, as previously reported, there was a lack of individual skill across the spectrum of the research process [42]. A further barrier to research participation and development of these missing skills is individual clinicians fear of making mistakes, with almost half of respondents being intimidated by research and having a ‘*fear of getting it wrong’.* These feelings of guilt and fear risk overshadowing recognition of the benefits to an individual’s own career development, and are a barrier that needs to be addressed in RCB strategies.

Not all staff are motivated to pursue a clinical academic career, or even undertake research, as demonstrated by a high proportion of respondents (38%) wanting to achieve a work-life balance instead as a barrier to research participation. Conversely, a primary motivator to engage in research was career advancement 67% (n=63), with the opportunity to develop outside a management career being further highlighted in the focus groups. However, it is unclear if this is a strong enough motivator in isolation, and sufficient to overcome the desire for a work-life balance. Additionally, job satisfaction (72% (n=67) and a *“desire to keep the brain stimulated”* (53%, n=49) and *“prove a theory/hunch”* (41%, n=38) were primary motivators. This exploratory nature of research with the potential to make a real life contribution to clinical practice is exciting and rewarding for staff to pursue [43]. Currently, it is unclear if there are common characteristics of successful clinical academics AHPs, however given the enduring and competitive nature of clinical academic careers, individual motivation will be key, and it is unlikely to be desired as a career path by the majority of AHPs [10]. Therefore RCB strategies also need to be tailored for the desired levels of research participation outside clinical academic careers.

Research influences clinical practice at an individual level by supporting and facilitating evidence-based practice. In our evaluation, participants appear to disproportionately place less importance and value on themselves than they do on teams and the department in the context of influencing research participation, capacity and capability. The findings from our evaluation will guide the development of our local RCB strategy which will address the barriers to research participation at a departmental, team and individual level. Future research should focus on establishing the role of the individual clinician in influencing opportunities and clinical academic careers and research participation.

### Strengths and limitations

This evaluation is limited by a low survey response rate at 33.8%, although this is higher than other previously published studies using the RCC tool [10, 42]. The comparatively favourable survey response rate may have been falsely elevated by a relatively high proportion of respondents currently involved in research activity, which accounted for 46%. Another potential limitation is the minimal personal information collected compared to previous studies which makes it more difficult to identify challenges within specific groups of similar attributes. Similarly, this service evaluation was conducted at a single department so this may limit transferability to other AHP departments due to varying management and clinical structures.

However, this evaluation has several important strengths. To the authors’ knowledge, this is the first mixed methods evaluation of AHP research capacity and culture that specifically serves as a precursor to developing a targeted local RCB programme. The focus of this future RCB programme is a large AHP department where research activity has historically been very low compared to other professional groups at the same organisation. Contributing to this is the limited supporting infrastructure in terms of established clinical academic career pathways and coordinated support for training and fellowship award applications. The future success of the RCB programme will, in part, be determined by the barriers and opportunities identified through this evaluation.

## Conclusions

In seeking to understand AHP research capacity and culture, this mixed methods evaluation finds a strong appetite and management support for research activity and evidence-based practice. However, multiple barriers cited as inhibitory to research activity and output were identified across all three domains of the RCC survey and were reflected within the focus groups. AHPs recognise the challenges to, and opportunities for research within the department and team domains, but less so their role and responsibilities as individuals. Future research should pay equal attention to understanding the role and influence at individual level on RCB strategies.

Mixed methods, research capacity, allied health professional, research culture, research activity.

## Supplementary Information

**Additional file 1.**

**Additional file 2.**

**Additional file 3.**

## Data Availability

All data generated or analysed during this study are included in this published article (and its supplementary information files).
