# Supplementary material for: Exploring research capacity and culture of allied health professionals: a mixed methods evaluation
Source: BMC Health Serv Res. 2022 Jan 17;22:85. doi: 10.1186/s12913-022-07480-x (PMC8764821; doi:10.1186/s12913-022-07480-x)
Supplement: Supplementary file 2 — Additional file 2. [file 12913_2022_7480_MOESM2_ESM.docx]

**A mixed methods evaluation of the Therapies Clinical Service Unit Research Capacity in Context.**

**STAFF FOCUS GROUP TOPIC GUIDE**

| Ulysses ID | 6309 |
| --- | --- |

**INTERVIEW INFORMATION**

| Ulysses ID | 6309 |
| --- | --- |
| Date of focus group |  |
| Number of staff attending |  |
| Research staff |  |

**WITHDRAWL FROM STUDY**

| STAFF MEMBER REQUESTED WITHDRAWAL FROM STUDY |  |
| --- | --- |
| DATA DESTROYED |  |

**INTERVIEW TOPIC GUIDE**

**AIM**

The aim of these focus groups is to further explore the results of the Research Capacity in Context survey, to identify key areas to address in the development of a Therapies Clinical Service Unit Research and Development strategy.

**CORE THEMES TO EXPLORE**

1. Opinions of increasing research capacity
2. Research career pathway structure
3. Perceived support required to increase research capacity

**INTERVIEW SCHEDULE**

Introduction to be read to participants

***“Thank you for taking part in this Focus Group. During the interview we explore the results of the Research Capacity in Context Survey in more detail. We will discuss how confident you feel both in your own research abilities and the support of your team and the Therapies CSU in enabling you to be more involved in research.***

***This session will be recorded by the research team, transcribed and any identifying data removed. You will not be identified in any future publication of this service evaluation but your quotes may be used to illustrate staff views on the subject. You may stop the interview at any time without giving us an explanation.”***

**QUESTIONS AND PROMPTS**

1. **Can you talk to me about any research experience that you may have?**
2. Did you do any research as part of your degree?
3. Have you attended any journal clubs/conferences/training?
4. Is there any research being undertaken in your clinical area?
5. **Do you think there are any benefits to increasing the research capacity within the Therapies CSU?**
6. Contribution to evidence based practice.
7. Patient experience.
8. Career development/links with HEI.
9. Job satisfaction/retention.
10. **What would be your vision of a research career pathway within the Therapies CSU?**
11. Initial exposure (internship/fellowship).
12. Clinical academic posts.
13. Joint posts with HEI.
14. Qualifications.
15. **What research training would you like from the Therapies CSU?**
16. Specific research skills (methodology, data analysis etc).
17. Sourcing funding and writing applications.
18. Preparing manuscripts/abstracts/posters/presentations.
19. Reading and analysing research/journal club.
20. **What support would you like from a research supervisor or mentor?**
21. Availability.
22. Expertise.
23. Communication.
24. Direction.
25. **How else would you like the Therapies CSU to support you in becoming more research active?**
26. Time (How? Amount? Protected?).
27. Funding (Courses? Research costs? Dissemination costs?).
28. Grant/fellowship applications.
29. Flexible working.

General Impressions from the interview

*Significant non-verbal cues, particular questions or thoughts raised that stood out, any changes to interview schedule that should be considered?*

Context of interview

*What was going on today that may have affected interview? Time pressures, sources of bias, reflexive reflections*

Additional reflections
