# Supplementary material for: Exploring research capacity and culture of allied health professionals: a mixed methods evaluation
Source: BMC Health Serv Res. 2022 Jan 17;22:85. doi: 10.1186/s12913-022-07480-x (PMC8764821; doi:10.1186/s12913-022-07480-x)
Supplement: Supplementary file 3 — Additional file 3. [file 12913_2022_7480_MOESM3_ESM.docx]

**Supplementary Material - Tables**

Table S1. Research activity

| **Item** | **Please indicate any research activity you are currently involved with (select as many as apply)** | **n (%)** |
| --- | --- | --- |
| i | Writing a research report, presentation or paper for publication | 19 (20) |
| ii | Writing a research protocol | 4 (4) |
| iii | Submitting an ethics application | 3 (3) |
| iv | Collecting data e.g. surveys, interviews | 25 (27) |
| v | Analysing qualitative research data | 12 (13) |
| vi | Analysing quantitative research data | 12 (13) |
| vii | Writing a literature review | 10 (11) |
| viii | Applying for research funding | 7 (8) |
| ix | Not currently involved with research | 50 (54) |
| x | Other | 5 (5) |
| xi | Research related activities as part of your role description | 29 (31) |
| **Item** | **What provisions are made for you to conduct research as part of your role? (select as many as apply)** | **n (%)** |
| i | Access to software | 1 (1) |
| ii | Research supervision | 5 (5) |
| iii | Time | 6 (6) |
| iv | Research funds | 1 (1) |
| v | Administrative support | 1 (1) |
| vi | Training | 5 (5) |
| vii | Library access | 16 (17) |
| viii | Other | 2 (2) |
| **Item** | **Please indicate if you have completed any of the following research activities in the past 12 months (select as many as apply)** | **n (%)** |
| i | Secured research funding | 5 (5) |
| ii | Co-authored a paper for publication | 12 (13) |
| iii | Presented research findings at a conferences | 13 (14) |
| iv | No research activity completed | 58 (62) |
| v | Other | 17 (18) |

Table S2. Barriers to undertaking research

| **Item** | **What are the barriers to research for you personally? (select as many as apply)** | **n (%)** |
| --- | --- | --- |
| i | Lack of time for research | 74 (80) |
| ii | Lack of suitable backfill | 50 (54) |
| iii | Other work takes priority | 65 (70) |
| iv | Lack of funds for research | 46 (49) |
| v | Lack of support from management | 22 (24) |
| vi | Lack of access to equipment for research | 35 (38) |
| vii | Lack of administrative support | 37 (40) |
| viii | Lack of software for research | 33 (35) |
| ix | Isolation | 15 (16) |
| x | Lack of library/internet access | 6 (6) |
| xi | Not interested in research | 8 (9) |
| xii | Other personal commitments | 15 (16) |
| xiii | Desire for work/life balance | 35 (38) |
| xiv | Lack of a co-ordinated approach to research | 32 (34) |
| xv | Lack of skills for research | 59 (63) |
| xvi | Intimidated by research language | 37 (40) |
| xvii | Intimidated by fear of getting it wrong | 42 (45) |
| xviii | Other | 8 (9) |

Table S3. Motivators to undertaking research

| **Item** | **What are the motivators to do research for you personally? (select as many as apply)** | **n (%)** |
| --- | --- | --- |
| i | To develop skills | 81 (87) |
| ii | Career advancement | 63 (67) |
| iii | Increased job satisfaction | 67 (72) |
| iv | Study or research related scholarships available | 33 (35) |
| v | Dedicated time for research | 44 (47) |
| vi | Research written into role description | 22 (24) |
| vii | Colleagues doing research | 30 (32) |
| viii | Mentors available to supervise | 46 (49) |
| ix | Research encouraged by managers | 40 (43) |
| x | Grant funds | 29 (31) |
| xi | Links to universities | 46 (49) |
| xii | Forms part of Post Graduate study | 30 (32) |
| xiii | Opportunities to participate at own level | 43 (46) |
| xiv | Problem identified that needs changing | 51 (55) |
| xv | Desire to prove a theory/hunch | 38 (41) |
| xvi | To keep the brain stimulated | 49 (53) |
| xvii | Increased credibility | 49 (53) |
| xviii | Other | 6 (6) |
